# Perineural Dexamethasone to Improve Postoperative Analgesia with Peripheral Nerve Blocks: A Meta-Analysis of Randomized Controlled Trials

**DOI:** 10.1155/2014/179029

**Published:** 2014-11-18

**Authors:** Gildasio S. De Oliveira, Lucas J. Castro Alves, Autoun Nader, Mark C. Kendall, Rohit Rahangdale, Robert J. McCarthy

**Affiliations:** Department of Anesthesiology, Feinberg School of Medicine, Northwestern University, 241 East Huron F5-704, Chicago, IL 60611, USA

## Abstract

*Background*. The overall effect of perineural dexamethasone on postoperative analgesia outcomes has yet to be quantified. The main objective of this quantitative review was to evaluate the effect of perineural dexamethasone as a nerve block adjunct on postoperative analgesia outcomes.* Methods*. A systematic search was performed to identify randomized controlled trials that evaluated the effects of perineural dexamethasone as a block adjunct on postoperative pain outcomes in patients receiving regional anesthesia. Meta-analysis was performed using a random-effect model.* Results*. Nine randomized trials with 760 subjects were included. The weighted mean difference (99% CI) of the combined effects favored perineural dexamethasone over control for analgesia duration, 473 (264 to 682) minutes, and motor block duration, 500 (154 to 846) minutes. Postoperative opioid consumption was also reduced in the perineural dexamethasone group compared to control, −8.5 (−12.3 to −4.6) mg of IV morphine equivalents. No significant neurological symptoms could have been attributed to the use of perineural dexamethasone.* Conclusions*. Perineural dexamethasone improves postoperative pain outcomes when given as an adjunct to brachial plexus blocks. There were no reports of persistent nerve injury attributed to perineural administration of the drug.

## 1. Introduction

Regional anesthesia has been commonly used as a strategy to mitigate postoperative pain in surgical patients [1–3]. Peripheral nerve blocks are particularly important in the ambulatory surgical setting since patients in that setting do not have access to potent intravenous analgesics in order to manage postoperative pain [4, 5]. However, a major limitation of peripheral nerve blocks can be a limited duration of analgesia [6, 7]. In order to circumvent that limitation, peripheral nerve catheters that offer continuously delivery of local anesthetics have been proposed as an efficient method of postoperative analgesia [8, 9]. Nevertheless, peripheral nerve catheters are costly and can be cumbersome to manage in the outpatient surgical setting [10, 11].

Dexamethasone is a systemic glucocorticoid commonly used to reduce postoperative nausea/vomiting pain and to improve quality of recovery after surgery [12–14]. Recently, several studies have examined the use of perineural dexamethasone in order to prolong analgesic duration of peripheral nerve blocks with variable results. However, the aggregated effect of perineural dexamethasone on analgesia outcomes has yet to be quantified. In addition, the safety of perineural dexamethasone also needs to be further examined [15].

The main objective of the current investigation was to evaluate the effect of perineural dexamethasone on analgesia outcomes along with peripheral nerve blocks. We also sought to examine safety concerns related to the use of perineural dexamethasone.

## 2. Methods

This quantitative systematic review was conducted following the guidelines of the PRISMA statement [16].

### 2.1. Systematic Search

Published reports of randomized trials evaluating the effects of perineural dexamethasone on postoperative anesthesia/analgesia were searched using the National Library of Medicine's PubMed database, Embase, the Cochrane Database of Systematic Reviews, and Google Scholar inclusive to August 15, 2013. Free text and MeSH terms “dexamethasone,” “nerve,” “block,” “regional,” “surgery,” “analgesia,” and “anesthesia” were used individually and in various pairwise combinations. No language restriction was used. An attempt to identify additional studies not found by the primary search methods was made by reviewing the reference lists from identified studies. No search was performed for unpublished studies. This initial search yielded 164 studies.

### 2.2. Selection of Included Studies

The study's inclusion and exclusion criteria were established before the systematic search. Two authors (Gildasio S. De Oliveira and Lucas J. Castro Alves) independently evaluated the abstract and results of the articles obtained by the initial search. Articles that were clearly not relevant based on our inclusion and exclusion criteria were excluded at this phase. Disagreements on inclusion of the articles were resolved by discussion among the evaluators. If an agreement could not be reached, the dispute was resolved with the help of a third investigator (Autoun Nader).

### 2.3. Inclusion and Exclusion Criteria

We included randomized controlled trials that examined perineural administration of dexamethasone for peripheral nerve blocks with the perineural administration of an inactive (placebo) control group. Trials reporting on the effects of dexamethasone on neuraxial blocks were excluded. Trials evaluating the effect of perineural dexamethasone in animals were also excluded. Studies containing the concurrent use of perineural drug were excluded if a direct comparison of dexamethasone and control could not be established. Volunteer studies in which subjects did not undergo surgical procedures were also excluded. Included studies had to report on clinical outcomes such as duration and/or onset of anesthesia/analgesia. No minimum sample size was required for inclusion in the meta-analysis.

### 2.4. Validity Scoring

Two authors (Gildasio S. De Oliveira and Lucas J. Castro Alves) independently read the included reports and assessed their methodological validity using a modified Jadad five-point quality scale [17]. The scale evaluates the study for the following: randomization, double blind evaluation, concealment of study group to evaluator, valid randomization method, and completeness of data at follow-up. As only randomized trials were included in the analysis, the minimum possible score of an included trial was 1 and the maximum was 5. Trials were not excluded or weighted in the analysis based on quality assessment scores.

### 2.5. Data Extraction

Two authors (Gildasio S. De Oliveira and Lucas J. Castro Alves) independently evaluated the full manuscripts of all included trials and performed data extraction using a data collection form specifically developed for this quantitative review.

Data extracted from trials included perineural dexamethasone dose, sample size, number of subjects in treatment groups, type of procedure, type of regional block, volume and dose of local anesthetics, analgesia duration (minutes), motor block onset and duration, cumulative opioid consumption, late pain (24 hours), and adverse events related to the regional anesthesia technique. Postoperative opioid consumption was converted to the equivalent dose of intravenous morphine [18]. Visual analog scale or numeric rating scale of pain was converted to a 0–10 numeric rating scale.

Data were initially extracted from tables. For data not available in tables, the data were abstracted from available figures. Dichotomous data on the presence or absence of adverse effects were extracted and converted to incidence while continuous data was recorded using mean and standard deviation. Data presented only as median and range was converted to means and standard deviation using previously described methodology [19]. The most conservative value was used when the same outcome was reported more than one time for a determined period.

### 2.6. Definition of Relevant Outcome Data

#### 2.6.1. Primary Outcomes

These included analgesia time (time in minutes to the first request of analgesia and/or reported pain), cumulative opioid consumption, early pain (<4 hours), and late pain (24 hours).

#### 2.6.2. Secondary Outcomes

These included oOnset and duration of motor block, onset of sensory block, and nerve block complications.

### 2.7. Meta-Analyses

The weighted mean differences (WMD) with 99% confidence interval (CI) were determined and reported for continuous data. For dichotomous data (adverse effects), the Peto odds ratio (to account for the potential of zero counts in the cells for low frequency outcomes) and 99% CI are reported. A significant effect compared to control required that the 99% CI for continuous data did not include zero and, for dichotomous data, the confidence interval did not include 1.0. Due to the inclusion of different procedures, we used a random effects model in an attempt to generalize our findings to studies not included in our meta-analysis. Although more computationally intensive, the random effects model has an advantage to the fixed effect model since it does not rely on the assumption that a true effect size is exactly the same in all combined studies [20]. Publication bias was evaluated by examining asymmetric funnel plots using Egger's regression test [21, 22]. A one sided *P* < 0.05 was considered as an indication of an asymmetric funnel plot. A file drawer analysis described by Rosenthal was performed in the case of an asymmetric funnel plot [23]. The test estimates the lowest number of additional studies that if they would become available would reduce the combined effect to nonsignificance assuming the average *z*-value of the combined *P* values of these missing studies would be 0.

Heterogeneity of the included studies was considered to be present if the *I* ^2^ statistic was greater than 30%. Further analysis was planned a priori to explore relevant heterogeneity. Subgroup analysis was performed to investigate the effect of type of regional block and local anesthetic on the effects of examined outcomes. A *Q* statistic was used to compare the effects between subgroups. The proportion of the total variance explained by the covariates (*R* ^2^) was calculated by dividing random effects pooled estimates of variance (Tau squared) within studies by total variance (total Tau squared). The value obtained was then subtracted from 1. When values fall outside the range of 0 to 100%, they were set to the closest value (0% or 100%).

A metaregression analysis was performed to evaluate the presence of a linear association between the perineural dexamethasone dose and the effect size on the analgesia duration [24]. Analysis was performed using Comprehensive Meta-Analysis software version 2 (Biostat, Englewood, NJ) and STATA version 11 (College Station, Texas, USA).

## 3. Results

**Figure 1:**
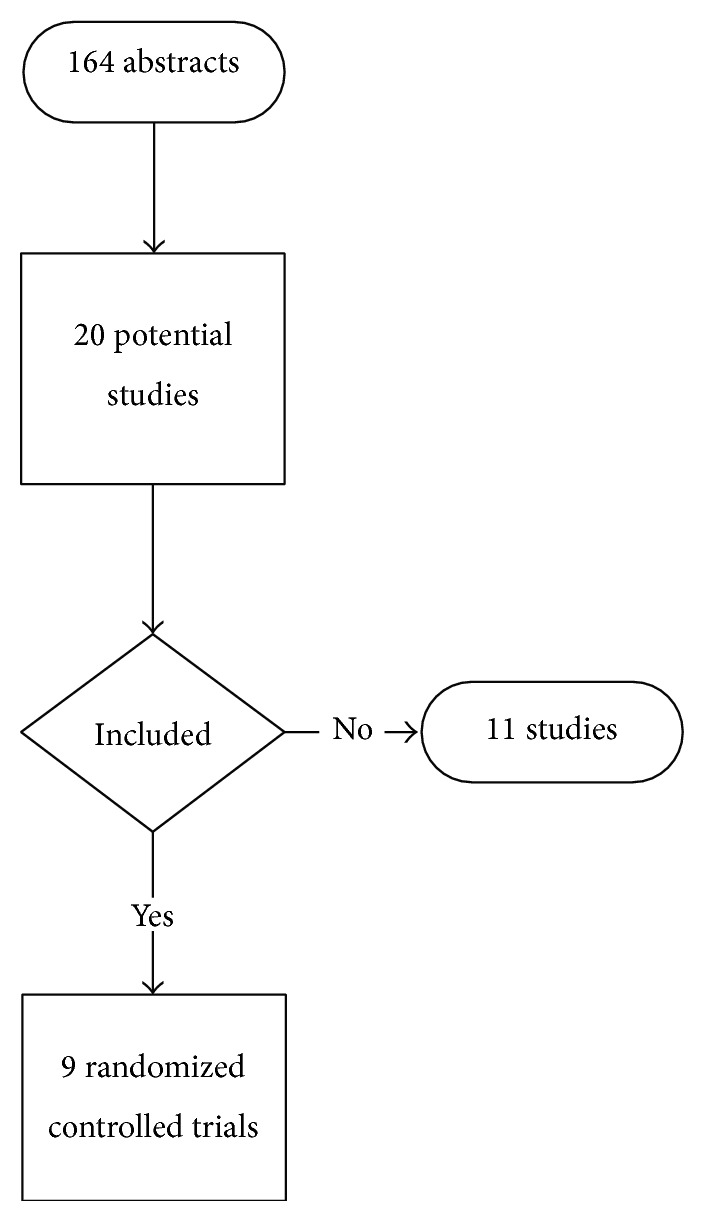
Flow chart outlining retrieved, excluded, and evaluated randomized controlled trials. Some trials evaluated multiple doses of dexamethasone.

Of the 164 initially evaluated abstracts, 20 studies were initially selected (Figure 1). Eleven studies were subsequently excluded: five studies were not randomized trials [25–29], three studies evaluated systemic dexamethasone [30–32], two studies did not evaluate peripheral blocks [33, 34], and one study did not provide a direct comparison [35]. The characteristics of included studies are listed in Table 1. The evaluated trials included data from 730 subjects and were published between 2006 and 2013 [36–44]. The median (IQR) number of patients in the included studies receiving perineural dexamethasone was 30 (24 to 49). The median (IQR) modified Jadad scale score was 4 (4 to 5). The trials tested the administration of perineural dexamethasone to patients undergoing upper extremity blocks for orthopedic procedures.

### 3.1. Analgesia Duration (the First Request for Pain Medications and/or Report of Pain in Minutes)

**Figure 2:**
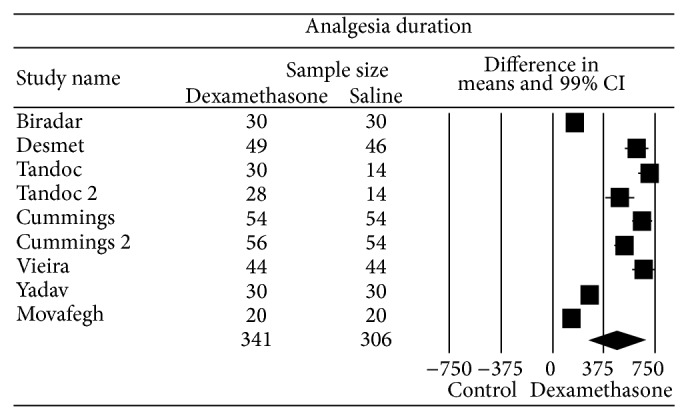
Pooled data evaluating the effect of perineural dexamethasone on analgesia duration compared to control. Data evaluated using a random effects model. Point estimate (99% CI) for overall effect was 473 (264 to 682) minutes. Weighted mean difference for individual study represented by square on forest plot with 99% confidence interval of the difference shown as solid line. Larger sized square and thicker 99% confidence interval line denote larger sample size. The diamond represents the pooled estimate and uncertainty for the effects of perineural dexamethasone compared to control.

The overall effect of seven studies (nine comparisons) [36–42] that examined perineural dexamethasone on analgesia duration compared to control favored dexamethasone with a weighted mean difference (99% CI) of 473 (264 to 682) minutes (Figure 2). Two studies contained two independent comparisons that were included in the analysis [38, 39]. The funnel plot was asymmetric suggesting the presence of publication bias; however, Rosenthal analysis suggested that it would be required to identify 6337 missing studies in order to change the analysis. Heterogeneity was high (*I* ^2^ = 93) but 22% of the total variance was explained by different blocks (interscalene versus supraclavicular or axillary). The overall effect of perineural dexamethasone on analgesia duration was increased when a subgroup analysis was performed including only interscalene blocks, WMD (99% CI) of 615 (528 to 701) min compared to control. There was no evidence for an asymmetric funnel plot (*P* = 0.48).

Metaregression analysis did not detect a linear association between perineural dexamethasone dose and duration of analgesia (*P* = 0.92).

### 3.2. Cumulative Opioid Consumption

**Figure 3:**
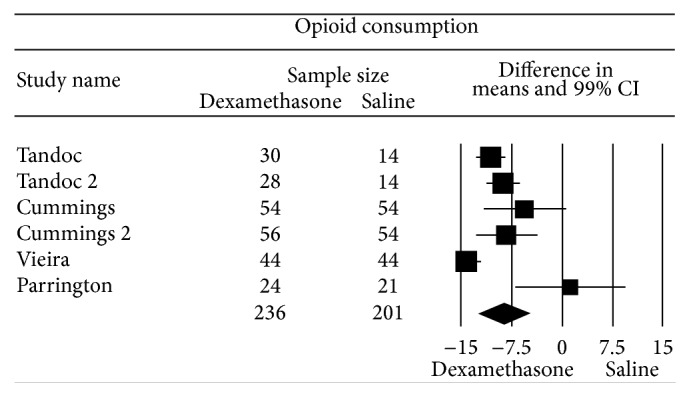
Pooled data evaluating the effect of perineural dexamethasone on postoperative opioid consumption compared to control. Data evaluated using a random effects model. Point estimate (99% CI) for overall effect was −8.5 (−12.3 to −4.6) mg of IV morphine equivalents. Weighted mean difference for individual study represented by square on forest plot with 99% confidence interval of the difference shown as solid line. Larger sized square and thicker 99% confidence interval line denote larger sample size. The diamond represents the pooled estimate and uncertainty for the effects of perineural dexamethasone compared to control.

The aggregated effect of four studies (six comparisons) [38–40, 43] that evaluated perineural dexamethasone on opioid consumption favored perineural dexamethasone compared to control, WMD (99% CI) of −8.5 (−12.3 to −4.6) mg of IV morphine equivalents (Figure 3). Funnel plot was asymmetric suggesting the possibility of publication bias (*P* = 0.04). Heterogeneity was high (*I* ^2^ = 86) and could not be explained by the type of the regional block or local anesthetic used. In contrast, the effect of perineural dexamethasone on postoperative opioid consumption was detected when bupivacaine and/or ropivacaine was used, WMD (95% CI) of −10.8 (−12.0 to 9.6) mg of IV morphine equivalents, but not when lidocaine was used, 1.3 (−6.8 to 9.4) mg of IV morphine equivalents, *P* < 0.001.

### 3.3. Late Pain (24 hours)

The overall effect of three studies [40, 43, 44] examining the effect of perineural dexamethasone on late pain did not show a beneficial effect of dexamethasone compared to control, WMD (99% CI) of −0.03 (−4.2 to 4.1). Heterogeneity was high (*I* ^2^ = 96) and it could not be explained by the type of the regional block or local anesthetic used.

### 3.4. Early Pain (<4 hours)

Only one study evaluated the effect of perineural dexamethasone on early pain suggesting a minor effect of perineural dexamethasone compared to control, WMD (99% CI) of −0.4 (−0.6 to −0.2) [44]. The study evaluated the use of perineural dexamethasone for patients receiving an interscalene brachial plexus block with levobupivacaine.

### 3.5. Block Onset (Sensory and Motor)

The aggregated effect of four studies [36, 41–43] that examined perineural dexamethasone on sensory block onset did not demonstrate a benefit of perineural dexamethasone compared to control, WMD (99% CI) of −0.6 (−2.9 to 1.5) min. Heterogeneity was high (*I* ^2^ = 77) and it could not be explained by the type of the regional block or type of local anesthetic used.

The combined effect of four studies [36, 41–43] that examined the effect of perineural dexamethasone compared to control on the onset of motor block did not detect a benefit of perineural dexamethasone relative to a large confidence interval, WMD (99% CI) of –1.0 (−3.1 to 1.1). Heterogeneity was high (*I* ^2^ = 77) and it could not be explained by the type of the regional block or type of local anesthetic used.

### 3.6. Motor Block Duration

**Figure 4:**
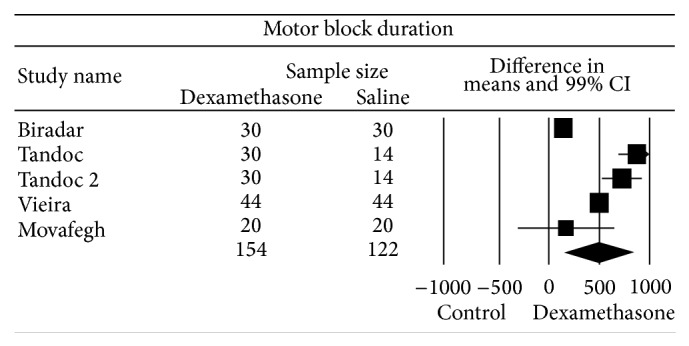
Pooled data evaluating the effect of perineural dexamethasone on motor block duration compared to control. Data evaluated using a random effects model. Point estimate (99% CI) for overall effect was 500 (154 to 846) min. Weighted mean difference for individual study represented by square on forest plot with 99% confidence interval of the difference shown as solid line. Larger sized square and thicker 99% confidence interval line denote larger sample size. The diamond represents the pooled estimate and uncertainty for the effects of perineural dexamethasone compared to control.

The overall effect of four studies (five comparisons) [36, 38, 40, 42] evaluating perineural dexamethasone compared to control on motor block duration favored dexamethasone, WMD (99% CI) of 500 (154 to 846) min (Figure 4). One study had two independent comparisons that were included in the analysis [38]. Egger's regression did not suggest the presence of an asymmetric funnel plot (*P* = 0.1). Heterogeneity was high (*I* ^2^ = 98) but it could be largely explained (64%) by studies that evaluated the effect of perineural dexamethasone on axillary/supraclavicular blocks as opposed to the interscalene block. The effect of perineural dexamethasone on motor block duration was greater for blocks performed with bupivacaine and/or ropivacaine, WMD (95% CI) of 696 (378 to 1015) compared to lidocaine, WMD (95% CI) of 155 (128 to 181).

#### 3.6.1. Safety Analysis

The majority of studies did not report any complications related to the use of perineural dexamethasone on peripheral nerve block. One study reported one episode of hypoesthesia in the dexamethasone group 4 months after surgery and none in the control group [37]. One study reported a greater but not statistically significant difference of tingling/numbness in the dexamethasone group (40%) compared to the saline group 26(%) two weeks after the operation.

## 4. Discussion

The most important finding of the current investigation was the substantially longer duration of analgesia in patients receiving dexamethasone as adjunct to brachial plexus blocks compared to control. Specifically for the interscalene approach, the use of dexamethasone with local anesthetics (ropivacaine or bupivacaine) prolonged analgesia duration for over 10 hours. In addition, the longer analgesic duration was accompanied by a lower consumption of postoperative opioids. Side effects were transient and could not be specifically attributed to the use of perineural dexamethasone. Taken together, our results suggest that perineural dexamethasone is an efficacious strategy to improve postoperative analgesia in subjects receiving brachial plexus blocks.

Our results are clinically important because the use of perineural dexamethasone as an adjunct to interscalene blocks provided analgesia duration for 24 hours in patients undergoing orthopedic surgery. Single injection nerve block with local anesthetics and dexamethasone may provide comparable analgesic results as peripheral nerve catheters for the first 24 hours after surgical procedures [9, 45, 46]. However, peripheral nerve catheters can have greater costs and complications such as catheter dislodgment or inadequate tip placement which can minimize the analgesic benefit of peripheral nerve catheters [8, 47, 48]. Nevertheless, future studies comparing single shot blocks using local anesthetics with dexamethasone to peripheral nerve catheters on analgesia outcomes are needed.

It was interesting to note the lack of benefit of perineural dexamethasone on late pain (24 hours) scores. Several reasons may explain this finding. First, it is possible that the similar pain scores in both groups were achieved due to a greater consumption of opioids in the control group compared to the perineural dexamethasone. Second, the duration of analgesia lasted approximately twenty four hours and the benefit may have decreased when the late pain outcome was measured. Lastly, the number of studies was not sufficient to detect an effect despite the apparent negative aggregate effect on late pain (WMD of −0.03).

We were unable to detect an association between perineural dexamethasone dose and effect size on the evaluated outcomes using metaregression analysis. It is possible that lower doses of dexamethasone may provide similar results on postoperative analgesia outcomes as the most commonly used 8 mg dexamethasone dose. In fact, a recent study suggested that even lower doses such as 1 mg may provide similar results on analgesia duration as greater dexamethasone doses [49].

We did not detect significant long lasting nerve complications that could be attributed to the use of perineural dexamethasone in our study population. One study reported one case of hypoesthesia in the dexamethasone group but the subject also had spinal disc herniation at the level of C4-5 which could have explained the symptoms [37]. Another study reported an increased but not statistically significant difference in the incidence of numbness and tingling in the dexamethasone group fourteen days after surgery. Since basic science studies have suggested the possibility of nerve toxicity by different perineural adjuncts, it is important that future studies provide longer follow-up evaluations of the included subjects [50–52].

It is also important to note that two recent studies have demonstrated similar benefits on analgesic duration of systemic compared to perineural dexamethasone for upper and lower extremity blocks [35, 37]. A potential advantage of perineural dexamethasone is the avoidance of undesirable side effects associated with the use of systemic dexamethasone [53–56]. Future studies evaluating the use of perineural dexamethasone on analgesia outcomes would benefit from the inclusion of a comparison group to evaluate the systemic dexamethasone administration.

Despite a benefit on postoperative analgesic duration and postoperative opioid consumption, the included studies did not evaluate the beneficial effect of perineural dexamethasone on more global recovery parameters [57, 58]. Inclusion of more global recovery outcomes is important since analgesic interventions may or may not demonstrate a benefit when more global recovery outcomes are evaluated [59–62]. Few studies have, in fact, been able to demonstrate a beneficial effect of regional anesthesia techniques on patient centered outcomes [63].

Our current quantitative review should only be interpreted in the context of its limitations. Some of our primary analysis revealed an asymmetric funnel plot suggesting the possibility of publication bias. Based on a Rosenthal analysis it is unlikely that the results would be changed by the detection of unpublished negative studies but the overall effect could be possibly reduced. In addition, publication bias was not detected when we performed a subgroup analysis of a single type of brachial plexus block. We also observed significant heterogeneity that could be partially explained by type of brachial plexus block and type of local anesthetic used. Lastly, we were unable to quantitatively estimate a potential harmful effect of perineural dexamethasone on postoperative nerve symptoms due to the low number of studies reporting those outcomes (*N* < 3).

In summary, perineural dexamethasone seems to improve analgesia duration and decrease opioid consumption when used as an adjunct to brachial plexus blocks. For interscalene blocks, perineural dexamethasone prolonged analgesia for more than ten hours. The included studies did not report on persistent nerve injury that could be attributed to the use of perineural dexamethasone. Perineural dexamethasone should be considered to improve postoperative analgesia in subjects receiving brachial plexus blocks for surgical procedures.

## Figures and Tables

**Table 1:** Summary of studies included in analysis.

Authors	Year of publication	Surgical specialty	Number of inadequate health literacy/total subjects	Health literacy instrument	Study design	Intervention	Outcome	Newcastle-Ottawa scale^*^ or modified Jadad score^†^
Ammar and Mahmoud [34]	2012	Orthopedics	86/144	REALM	Case-control	None	Medical information comprehension	4^*^

Fredrickson et al. [35]	2013	Pediatrics	30/79	Newest vital sign	Cross-sectional	None	Satisfaction with information	3^*^

Biradar et al. [36]	2013	Gynecology	102/201	Chew's screening items	Randomized controlled trial	Low literacy consent form	Consent form comprehension	3^†^

Desmet et al. [37]	2013	Orthopedics	15/15	S-TOHFLA	Focus groups	Pictograph based discharge instructions	None	—

Kim et al. [44]	2012	Ear, nose, and throat	3/8	S-TOHFLA	Cross-sectional	None	None	3^*^

Tandoc et al., [38]	2011	Vascular	70/152	REALM	Cross-sectional	None	None	3^*^

Cummings III et al., [39]	2011	Transplant	14/62	TOHFLA	Cohort	None	Access to kidney transplant	5^*^

Vieira et al., [40]	2010	Transplant	6/124	S-TOHFLA/ REALM-T	Cross sectional	None	Kidney function	5^*^

Parrington et al., [43]	2010	All ambulatory surgeries	40/332	S-TOHFLA	Cohort	None	Adherence to preoperative instructions	5^*^
